# Chest x-ray findings and temporal lung changes in patients with COVID-19 pneumonia

**DOI:** 10.1186/s12890-020-01286-5

**Published:** 2020-09-15

**Authors:** Liqa A. Rousan, Eyhab Elobeid, Musaab Karrar, Yousef Khader

**Affiliations:** 1 Department of Diagnostic Radiology and Nuclear Medicine, Jordan University of Science and Technology, PO BOX 3030, Irbid, 21100 Jordan; 2 Department of Emergency, Jordan University of Science and Technology, Irbid, Jordan; 3 Department of Public Health, Community Medicine, Jordan University of Science and Technology, Irbid, Jordan

## Abstract

**Background:**

Chest CT scan and chest x-rays show characteristic radiographic findings in patients with COVID-19 pneumonia. Chest x-ray can be used in diagnosis and follow up in patients with COVID-19 pneumonia. The study aims at describing the chest x-ray findings and temporal radiographic changes in COVID-19 patients.

**Methods:**

From March 15 to April 20, 2020 patients with positive reverse transcription polymerase chain reaction (RT-PCR) for COVID-19 were retrospectively studied. Patients’ demographics, clinical characteristics, and chest x-ray findings were reported. Radiographic findings were correlated with the course of the illness and patients’ symptoms.

**Results:**

A total of 88 patients (50 (56.8%) females and 38 (43.2%) males) were admitted to the hospital with confirmed COVID-19. Their age ranged from 3 to 80 years (35.2 ± 18.2 years). 48/88 (45%) were symptomatic, only 13/88 (45.5%) showed abnormal chest x-ray findings. A total of 190 chest x-rays were obtained for the 88 patients with a total of 59/190 (31%) abnormal chest x-rays. The most common finding on chest x-rays was peripheral ground glass opacities (GGO) affecting the lower lobes. In the course of illness, the GGO progressed into consolidations peaking around 6–11 days (GGO 70%, consolidations 30%). The consolidations regressed into GGO towards the later phase of the illness at 12–17 days (GGO 80%, consolidations 10%). There was increase in the frequency of normal chest x-rays from 9% at days 6–11 up to 33% after 18 days indicating a healing phase. The majority (12/13, 92.3%) of patients with abnormal chest x-rays were symptomatic (*P* = 0.005).

**Conclusion:**

Almost half of patients with COVID-19 have abnormal chest x-ray findings with peripheral GGO affecting the lower lobes being the most common finding. Chest x-ray can be used in diagnosis and follow up in patients with COVID-19 pneumonia.

## Background

An outbreak of severe cases of pneumonia from an unidentified origin emerged in Wuhan, China in December 31, 2019. The illness rapidly spread in China and in many other countries. In January 2020, the World Health Organization (WHO) declared it a pandemic [1]. A virus was identified and isolated from the epithelial cells of the respiratory system of infected individuals and was named as Severe Acute Respiratory Syndrome Coronavirus 2 (SARS-CoV-2) and the outbreak was named coronavirus disease (COVID-19) [2].

Coronaviruses are enveloped, positive-sense, single strand, non-segmented, and ribonucleic acid viruses that belong to the coronaviridae family [3]. The viruses have characteristic morphology under the electron microscope with presence of viral spike peplomers arising from the viral envelope giving a crown appearance [4]. The coronaviruses are widely distributed among humans and mammals [5]. Six coronaviruses are identified, four of which cause mild common cold symptoms, and two strains were responsible for Severe Acute Respiratory Syndrome (SARS) that began in southern China in 2003 and Middle East Respiratory Syndrome (MERS) that originated in Saudi Arabia in 2012 [6].

The most common symptoms of COVID-19 include fever, cough, dyspnea, fatigue, and myalgia, less common symptoms are sputum, hemoptysis, headache, and gastrointestinal symptoms [5].COVID-19 infection is confirmed in many countries by Reverse Transcription Polymerase Chain Reaction (RT-PCR) on nasopharyngeal and throat swabs, with a positive rate of 30–70% [7, 8]. Chest CT scan was found to be more sensitive than RT-PCR in confirming the diagnosis of COVID-19 reaching 98% [8]. Chest x-ray was found to have limited value in the initial diagnosis of COVID-19 with a sensitivity of about 69% [9, 10]. Patients with COVID-19 had typical radiological findings on chest imaging including multifocal and bilateral ground glass opacities and consolidations with peripheral and basal predominance. Septal thickening, bronchiectasis, pleural effusion, lymphadenopathy, and cavitation were less commonly seen [1, 6, 11–14].

The outbreak of COVID-19 began in March 2020 in Jordan. RT-PCR was used in the diagnosis and chest x-ray was used in the follow up of patients. Information on chest x-ray findings in patients with COVID-19 pneumonia is still limited in the literature and the majority of the reports described the lung changes on chest CT scan. This study aimed to report the chest x-ray findings in 88 patients with confirmed COVID-19 and to describe the temporal changes of the chest radiological findings throughout the disease course.

## Methods

### Study design

This is a retrospective study of laboratory confirmed COVID-19 patients who were admitted to the isolation wards in a tertiary teaching hospital between March 15 and April 20, 2020. The hospital is the largest tertiary center in the North part of Jordan and was the second largest isolation center during the pandemic. Admission criteria included positive RT-PCR on nasopharyngeal swabs in any individual with a history of contact with a confirmed COVID-19 patient, or any individual with recent history of travel. Patients were admitted to the isolation wards even before the onset of symptoms. Patients were discharged after two consecutive negative RT-PCR tests at least 72 h apart.

A structured form was used to extract the data from the electronic medical records. Data collected included sociodemographic characteristics, presenting symptoms, past medical history, and RT-PCR and chest radiographic findings. The study was approved by the Institutional Review Board at Jordan University of Science and Technology. The written consent was waived by the ethics committee.

### Image acquisition and analyses

All the chest x-rays were acquired as a digital radiograph in the anteroposterior projection using portable x-ray units in the isolation wards following local protocols. The chest x-rays were analyzed by two radiologists who were blinded to the presence or absence of symptoms followed by joint consensus. The radiographic features were diagnosed according to the Fleischner society glossary. Ground glass opacity (GGO) was defined as an increase in opacification of the lung which does not obscure the blood vessels and airways. Consolidation was defined as a homogenous opacification that obscures the blood vessels and airway walls. Reticulation was defined as a collection of innumerable small opacities in a linear pattern [15]. Presence of nodular consolidation, and pleural effusion were also recorded.

The distribution of the lung lesions was classified into: 1) right lung, left lung, or bilateral. 2) Peripheral predominant, central predominant, or diffuse. Demarcation was defined as halfway between lateral edge of the lung and the hilum. 3) Zonal distribution. The upper zone extends from the superior hilar markings to the apices of the lungs, the middle zone extends from the inferior hilar markings to the superior hilar markings, and the lower zone extends from the costophrenic sulcus to the inferior hilar markings.

A severity score was determined for each lung using the Radiographic Assessment of Lung Edema (RALE) score proposed by Warren et al. [16]. The score is determined by the involvement of each lung by consolidation or ground glass opacity from 0 to 4 (0 = no involvement; 1 = < 25%; 2 = 25–50%; 3 = 50–75%; 4 = > 75% involvement). The scores for each lung were summed to produce the final severity score.

Baseline and serial chest x-rays were reviewed and were compared to determine if there was progression, stability, or improvement of lung changes over the time course of the illness. The serial follow up chest x-rays were categorized according to the time of onset of symptoms: chest x-rays performed at 0–5 days, 6–11 days, 12–17 days, and over 18 days from onset of symptoms.

The chest x-rays were correlated with patients’ symptoms and RT-PCRs results. Onset of symptoms to time of positive chest x-ray as well as time interval between chest x-ray examinations and time interval between RT-PCR tests were obtained. In the case of asymptomatic patients, date of first positive RT-PCR was substituted for symptom onset.

### Statistical analysis

Data were analyzed using the IBM SPSS version 24. Categorical data were described using frequencies and percentages and continuous data were described using means and standard deviation. Chi-square test was used to compare percentages. A *p*-value of less than 0.05 was considered statistically significant.

## Results

### Patients’ characteristics

A total of 88 patients (50 (56.8%) females and 38 (43.2%) males) were admitted to the hospital with confirmed COVID-19 during the study period. The average (±SD) age was 35.2 ± 18.2 years (range 3–80 years). Forty-eight patients (54.5%) were symptomatic and 40 patients (45.5%) were asymptomatic. Cough and fever were the most frequent symptoms (33 and 17%, respectively). The most common co-morbidities among the patients were hypertension (15.9%) and diabetes (10.2%). The majority of the patients (96.6%) had a history of contact with infected individuals and 5.7% had history of travel overseas. The mean time from initial positive RT-PCR to negative RT-PCR was 13 ± 3 days (range 7–19 days). Table 1 shows patients’ demographic characteristics, clinical presentation, co-morbidities, and clinical outcomes.

**Table 1:** Patients’ demographics, characteristics, clinical presentation, co-morbidities, and clinical outcome (*n* = 88)

Characteristics	Number (%)
Sex	
Male	38 (43.2)
Female	50 (56.8)
Age (years), mean ± SD	35.24 ± 18.21
Travel History	5(5.7)
Contact History	85(96.6)
**Clinical Presentation**	
Symptomatic	48 (54.5)
Asymptomatic	40 (45.5)
**Symptoms**	
Fever	15 (17)
Headache	10 (11.4)
Cough	29 (33)
Shortness of breath	6 (6.8)
Sore throat	11 (12.5)
General weakness	7 (8)
Diarrhea	3 (3.4)
Vomiting	1 (1.1)
**Co-morbidities**	
Diabetes	9 (10.2)
Hypertension	14 (15.9)
Asthma	0 (0)
Ischemic heart disease	3 (3.4)
Malignancy	2 (2.3)
**Clinical outcomes at the end of study**	
Discharge	52 (59.1)
In admission	35 (39.8)
Died	1 (1.1)

### Chest x-ray features

A total of 190 chest x-rays were performed for the 88 patients; 88 chest x-rays as baseline, and 102 chest x-rays as follow up. Of the 88 patients, 13 (14.8%) demonstrated abnormalities on chest x-rays at some time point during their illness (ten patients at baseline and three developed abnormalities during the follow-up) with a total of 59/190 (31%) abnormal chest x-rays. Seventy-five (85%) patients had no chest x-ray abnormalities although they tested positive for COVID-19 by RT-PCR.

The mean time from initial positive chest x-ray to negative chest x-ray was 10.9 ± 3.6 days (range 6–14 days). Almost half (38/75, 50.7%) of the patients with normal chest x-ray were symptomatic and the majority (12/13, 92.3%) of patients with abnormal chest x-rays were symptomatic, there was a significant association between the chest x-ray findings and the symptoms (*P* = 0.005). Only one patient with positive chest x-ray findings remained asymptomatic throughout the course of the illness.

**Fig. 1:**
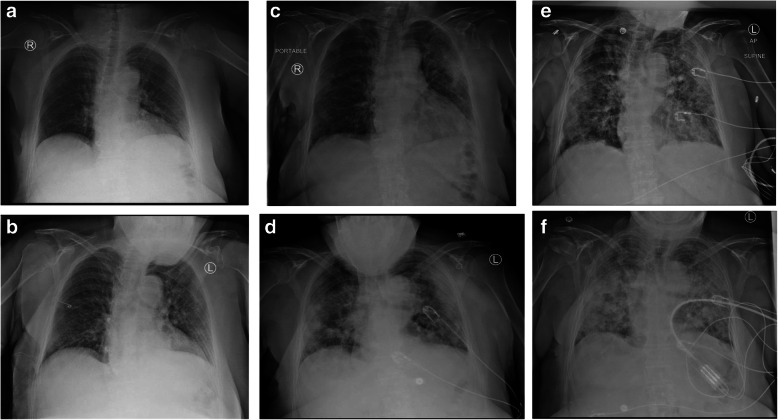
Series chest x-rays in an 80-year-old woman with COVID-19 pneumonia. **a** Chest x-ray obtained on illness day 5 showed peripheral GGO in the LLZ (score 1). **b** Chest x-ray obtained on illness day 7 showed increase extent of the GGO diffusely involving the left lung (score 4). **c** Chest x-ray obtained on illness day 11 showed increase extent of the GGO involving the right lung, with increase extent of consolidation involving the left lung diffusely (Total score 8). **d** Chest x-ray obtained on illness day 14 showed development of reticulations in both lungs with increase extent of involvement of the RUZ. (Total score 8). **e** Chest x-ray obtained on illness day 17 showed extensive bilateral consolidations mainly peripherally with increased reticulations (Total score 8). **f** Chest x-ray obtained on illness day 18 showed extensive consolidation involving both lungs diffusely (Total score 8). The patient died on illness day 18. (GGO: ground glass opacity. LLZ: left lower zone. RUZ: right upper zone)

**Fig. 2:**
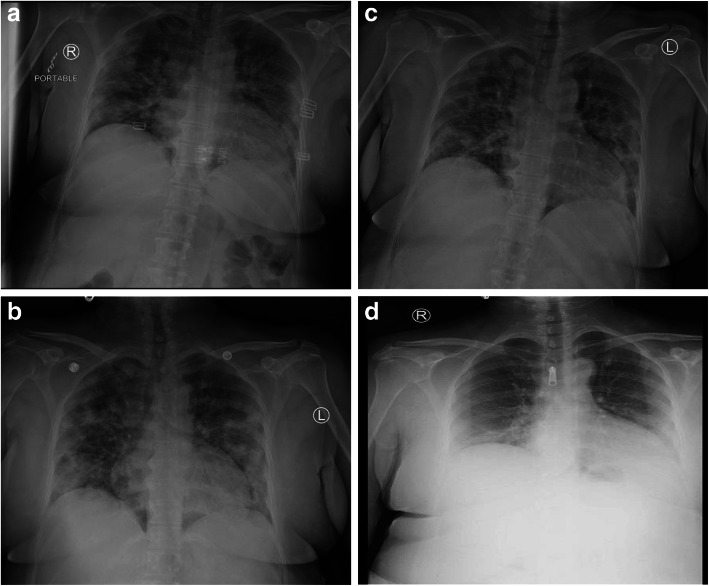
Series chest x-rays in a 49-year-old woman with COVID-19 pneumonia. **a** Chest x-ray obtained on illness day 1 showed bilateral central and peripheral (diffuse) GGO bilaterally (Total score 7, right 4 Vs left 3). **b** Chest x-ray obtained on illness day 5 showed peaking of the findings with diffuse patchy and nodular consolidations bilaterally (Total score 8). **c** Chest x-ray obtained on illness day 8 showed decrease in the degree of lung involvement with reduction in the overall severity score, however, there was development of reticulations in the upper zones (Total score 5 right 3 Vs left 2). **d** Chest x-ray obtained on illness day 15 showed the absorption phase with regression of the consolidations into peripheral GGO seen in the lower zones bilaterally with a total score of 2

During the study period, three patients (23%) progressed rapidly over an average period of 4 days with increase in the total chest x-ray severity score on average from 1 to 7. Only one elderly female patient (80 years) passed away at day 18 of onset of symptoms (Fig. 1). Nine patients (69%) showed improvement in the chest x-ray findings with almost complete resolution of the abnormalities (Fig. 2). The chest x-ray findings in one patient remained stable.

Baseline chest x-rays were done on average at day three from symptom onset. Only ten patients (11.3%) had abnormalities on their baseline chest x-ray, GGO was the only radiographic lung abnormality detected on the chest x-rays in all ten patients. Peripheral location of the opacities and right lower zone distribution were the most common locations (9/10 (90%) and 7/10(70%), respectively). Pleural effusion was found in the chest x-ray of one patient only (Table 2). Nine out of 10 (90%) patients had mild radiographic findings with total severity score of 1–2. Only one patient had a total severity score of seven (score in the right lung was 4 in the left lung was 3).

**Table 2:** Radiographic findings and distribution on baseline chest x-ray in 10 patients

Type of parenchymal opacity at CXR	Number (%)
Consolidation	0 (0)
Ground glass opacity	10(100)
**Distribution at CXR**	
Peripheral predominant	9 (90)
Perihilar predominant	0 (0)
Diffuse	1 (10)
Right lung	4 (40)
Left lung	3 (30)
Bilateral lungs	3 (30)
**Lobar Involvement**	
Right upper zone	1 (10)
Right middle zone	1 (10)
Right lower zone	7 (70)
Left upper zone	1 (10)
Left middle zone	2 (20)
Left lower zone	5 (50)
**Other features on chest x-ray**	
Pleural effusion	1 (10)

On serial follow up chest x-rays; GGO remained the most common lung abnormality pattern. At 0–5 days from onset of symptoms, the frequency of the GGO was 55% and consolidation was 20%.The rest of the chest x-rays (25%) were normal. At 6–11 days the percentage of x-rays with GGO and the consolidations increased to 70 and 30% respectively, with decrease in the number of normal chest x-rays (2/23 (9%)). One patient developed pleural effusion.

At 12–17 days, the consolidations regressed and the GGO increased (10 and 80%, respectively) with a mixed pattern of nodular consolidations and GGO in 17%. Reticulations developed within this phase comprising 8% of the abnormalities. The frequency of normal chest x-rays was zero in this group.

**Fig. 3:**
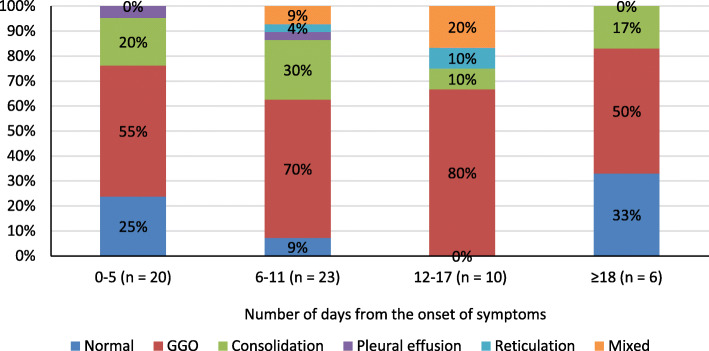
Temporal change of chest x-ray findings. Stacked-bar graph showed the distribution of the lung abnormalities on chest x-ray at various time points from symptom onset. GGO was the most frequent abnormality on initial x-rays, consolidation increased in frequency till the second week then regressed into GGO which again was more frequent on subsequent chest x-rays. Mixed pattern of GGO and nodular consolidation and reticulations were noted in the second week. Normal chest x-rays increased in frequency with time as patients showed clinical improvement. GGO = ground glass opacity

After 18 days, the lung abnormalities regressed (50% GGO and 17% consolidation), with increase in the frequency of normal chest x-rays (33%) indicating a healing phase. Figure 3 shows the distribution of lung abnormalities at different time intervals from onset of symptoms.

The spatial distribution of the radiographic lung changes increased throughout the course of the disease. Earlier in the disease (days 0–5) bilateral involvement was seen in 30%, exclusive unilateral involvement was observed in 5/20 (25%) on the right, and 4/20 on the left (20%).The lower zones were more frequently involved (55% right, 40% left). The lung abnormalities were seen predominantly in the periphery of the lungs.

At days 11–6 from onset of symptoms, the percentage of involvement of the lower zones increased and remained the most common (65% right lower zone, 52% left lower zone). The lung abnormalities extended from the periphery to the central giving a diffuse pattern in 25%. Exclusive involvement of the right lung was noted in the majority of the x-rays (40%). Bilateral involvement was noted in 35% of the x-rays.

At days 12–17 from onset of symptoms; involvement of the left lower zone predominated (80%). Bilateral involvement was most common at this stage (80%).

After 18 days from the onset of symptoms; the right upper and right middle zones were the last to recover (66 and 50% respectively). The frequency of involvement of the other lobes decreased with fewer findings seen centrally and complete resolution of the left lung.

**Fig. 4:**
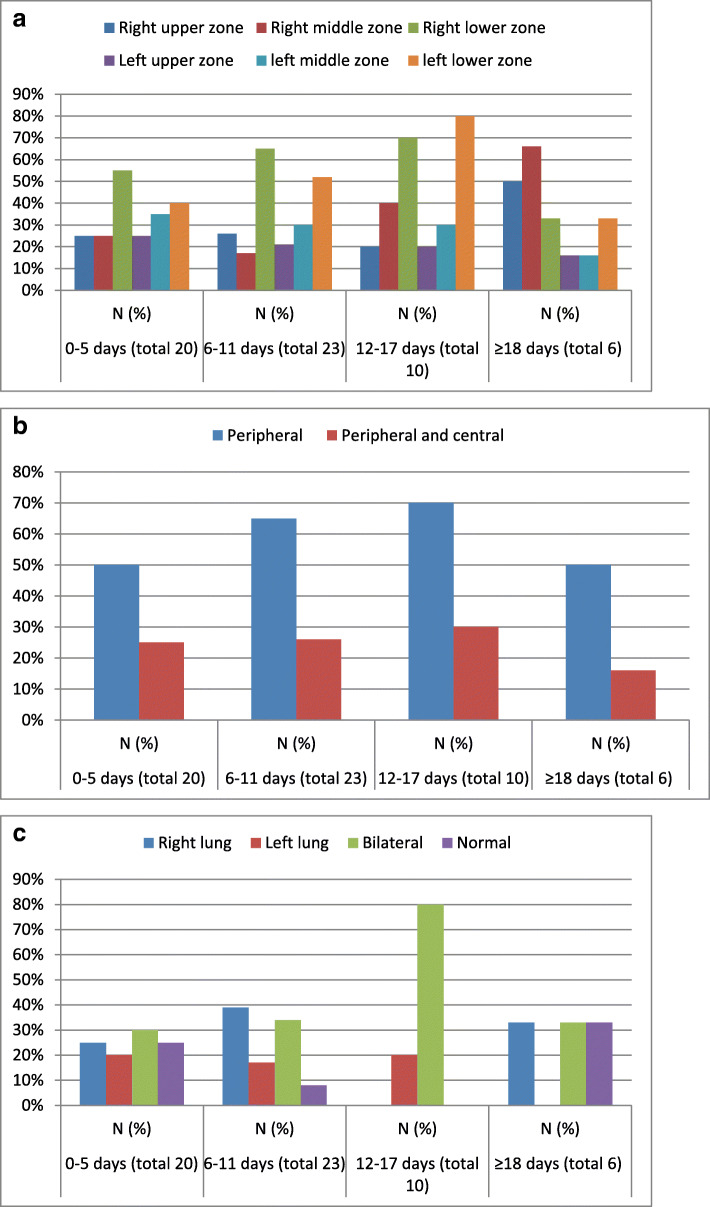
The spatial distribution of the lung changes at various time intervals from symptom onset. **a** Zonal distribution. The right lower zone remained the most frequently involved over time, the left upper and left middle zones were the least to be involved. **b** Horizontal distribution. The lung changes were more frequently seen in a peripheral distribution. Isolated central involvement of the lung changes was not observed in any of the chest x-rays. **c** Distribution according to side. Bilateral distribution of the lung changes was more common than unilateral involvement

The left middle and left upper zones were the least to be involved throughout the course of the illness. Exclusive involvement of the central parts of the lungs was not observed in any of the chest x-rays. The rate of normal chest x-rays decreased from 25% at 0–5 days to none at 12–17 days, then increased to 33% as patients showed recovery. The specific frequencies of the spatial distribution of the lung changes are summarized in Fig. 4.

**Fig. 5:**
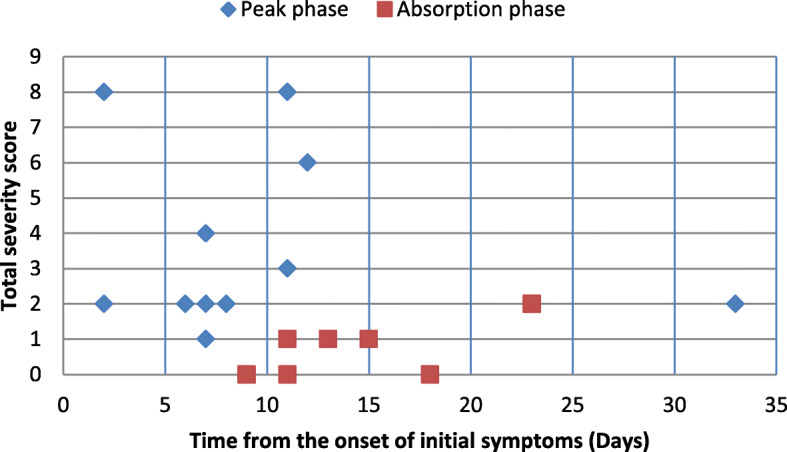
Temporal change of severity score. Scatter graph showed the maximum total severity score at the peak phase reaching at days 5–10 from onset of symptoms, with an average of severity score 3, (*n* = 13). The total severity score decreased over time as the chest x-ray findings regressed at days 10–15 from onset of symptoms (*n* = 9)

The highest severity score recorded was eight (of maximum possible score of eight). Peak severity score was reached at day 5–10 from symptom onset, known as the peak phase at which the median chest x-ray severity score was three. Nine out of 13 patients (69%) showed complete or near complete resolution of the chest x-ray findings which was reached at day 10–15 from symptom onset known as the absorption phase (Fig. 5).

## Discussion

RT-PCR was the first line of diagnosis in patients with COVID-19 in Jordan. In previous reports, chest CT scan was found to be a more sensitive diagnostic tool than RT-PCR even in asymptomatic patients reaching 98% [1, 7, 8]. However, many researchers found that patients with a positive RT-PCR may have a negative chest CT scan, and patients with a negative RT-PCR may have positive chest CT scan [4, 7, 12]. Chest x-ray was regarded an insensitive tool reaching 69% [8, 9, 14, 17]. The American College of Radiologists (ACR) and the Fleischner Society have suggested that imaging is not advised for patients who tested positive by RT-PCR who were asymptomatic or have mild symptoms, and CT scan should be reserved for patients with a progressive disease course [18, 19]. Due to the high infectious rate of COVID-19 virus; infection control in radiology departments becomes a challenge in the CT scan suite, therefore, the ACR has also recommended that portable chest x-ray may be considered to minimize the risk of cross infection [14, 18].

In our study, every patient had at least one chest x-ray done during their stay in the hospital, no chest CT scan was performed in any of the patients. Only one patient (1/88, 12.5%) with positive chest x-ray findings and positive RT-PCR remained asymptomatic throughout the illness. Asymptomatic patients with positive RT-PCR results and chest CT scan findings were reported in the literature [10, 20] and may be as a result of acquiring immunity from a previous infection or being in the healing phase [20]. Normal chest x-rays in RT-PCR positive patients was seen in 25% and 31% in previous reports [14, 21]. In our study, 85% of the patients who tested positive for COVID-19 had negative chest x-rays, 50% of them were asymptomatic the other half had mild symptoms. Identifying patients with COVID-19 positive RT-PCR is essential in containing the disease by isolating the patients to prevent further spread of the disease.

The most common symptom among our patients was cough followed by fever, which is the common presentation among patients with COVID-19 pneumonia worldwide [5, 22]. Three percent of our patients suffered from diarrhea which was described by the patients as the worst diarrhea ever experienced. Diarrhea was also an uncommon symptom in previously reported patients [5, 17, 23].

The most common chest x-ray finding in our patients was GGO in a peripheral distribution with bilateral lung involvement, there was a lower lobe predilection of the opacities, with the right lower lobe more common than the left lower lobe (70% vs. 50%). Our findings are in consensus with previous studies on chest x-ray and chest CT scans [4, 8, 11–13, 17, 21–26]. Only two patients had pleural effusion which is not a common finding on chest imaging [14, 27]. Two patients developed reticulations in the second week from the onset of symptoms, this finding was reported on chest CT scan [7, 23, 25, 28]. However, it was reported earlier in the course of the disease on chest x-ray in one large study [21].

The chest x-ray severity scores changed over time, peaking at day 5–10 of symptom onset with transformation of the GGO into focal areas of consolidations and into nodular consolidations. A phase of improvement of the findings with decrease in the size and number of the GGO/consolidation and lobes involved and regression of consolidations into GGO was observed in 69% of the patients at day 10–15 from onset of symptoms. The peak and the absorption phases in our study were observed to be earlier than those reported previously [peak phase range at days 6–15, absorption phase range at days 14–17] [1, 23–25].

Chest x-ray severity score was found in a previous report to be a predictive index of risk for hospital admission and intubation in patients with COVID-19 pneumonia [29], and mobile chest x-rays were found to be beneficial in the follow up of critically ill COVID-19 patients in another study [27]. In our study, the radiographic findings on chest x-ray in COIVD-19 pneumonia patients are consistent with the radiographic findings detected on chest CT scans and on chest x-rays in previous reports. Also, in our study the presence of symptoms correlated significantly with abnormal chest x-ray findings suggesting that chest x-ray may be helpful as an aiding tool in the diagnosis and follow up in patients with COVID-19 pneumonia.

The limitations of our study include small sample size of the patients with positive chest x-ray findings and short follow up period. In addition, the interval between the chest x-rays obtained was not uniform in all patients which may have led to undiagnosed abnormalities. And the lack of correlation between chest x-ray and chest CT scan findings.

## Conclusion

Almost half of the patients with COVID-19 had abnormal chest x-ray findings, GGO in a peripheral distribution with lower lobe predilection being the most common findings on chest x-ray. The radiographic findings peaked at day 5–10 of symptom onset reaching the highest severity score. Presence of symptoms correlated significantly with abnormal chest x-ray findings. Chest x-ray may be helpful as an aiding tool in the diagnosis and follow up in patients with COVID-19 pneumonia.

## Data Availability

Institutional policy does not allow sharing of data and materials publicly.

## Abbreviations

RT-PCR: Reverse transcription polymerase chain reaction
GGO: Ground glass opacities
